# Dose‐dependent hepatotoxicity of hydrogen peroxide in HepG2 cells and its modulation by CYP450 induction

**DOI:** 10.1002/2211-5463.70299

**Published:** 2026-06-28

**Authors:** Maren Jinks, Garth L. Maker, Emily C. Davies, Berin A. Boughton, Samantha Lodge

**Affiliations:** ^1^ Centre for Computational and Systems Medicine, Health Futures Institute, Harry Perkins Building Murdoch University Perth Australia; ^2^ Medical, Molecular and Forensic Sciences Murdoch University 90 South Street Murdoch Australia; ^3^ Leukaemia Translational Research Laboratory WA Kids Cancer Centre, The Kids Research Institute Australia Perth Australia; ^4^ Curtin Medical School Curtin University Perth Australia; ^5^ Department of Microbiology PathWest Laboratory Medicine Perth Australia; ^6^ La Trobe Institute for Sustainable Agriculture and Food, AgriBio La Trobe University Bundoora Australia

**Keywords:** cytochrome P450, HepG2, hydrogen peroxide, *in vitro* toxicology, metabolomics, model systems

## Abstract

*In vitro* liver models combined with metabolomics approaches offer promising alternatives to animal testing in toxicology. In this study, we investigated concentration‐dependent effects of hydrogen peroxide (H_2_O_2_) on the intra‐ and extracellular metabolome of HepG2 cells using ^1^H Nuclear Magnetic Resonance (NMR) spectroscopy. After cells were exposed to low, medium or high concentrations of H_2_O_2_, metabolomic analysis revealed a progressive increase in metabolic perturbation with rising toxin concentration. Significant alterations were detected in a limited subset of metabolites after low H_2_O_2_ exposure, and substantially broader disruptions occurred after medium or high H_2_O_2_ exposure, with most measured metabolites affected at the highest exposure level. To enhance metabolic competence, cells were pretreated with rifampicin to induce cytochrome P450 (CYP450) activity, which is typically low in HepG2 cells. Comparative analysis of rifampicin‐pretreated and untreated cells exposed to high H_2_O_2_ concentrations demonstrated disruption of multiple biochemical pathways, including energy metabolism, lipid metabolism and amino acid metabolism. Notably, rifampicin pretreatment attenuated the magnitude of metabolic perturbations, as reflected by reduced intracellular alterations and minimal changes in extracellular metabolite profiles. Furthermore, rifampicin‐treated cells exhibited metabolite signatures more consistent with human liver physiology *in vivo*, including increased glutathione and 2‐hydroxybutyrate levels. Collectively, these findings demonstrate that pretreatment with rifampicin prior to toxin exposure enhances the physiological relevance of HepG2‐based hepatotoxicity models and improves their potential to predict human liver responses. Moreover, the results highlight the sensitivity of NMR‐based metabolomics to detect toxin induced metabolic changes across a range of exposure concentrations.

## Graphical abstract

NMR metabolomics revealed concentration‐dependent metabolic perturbations in HepG2 cells exposed to H_2_O_2_. Rifampicin pretreatment enhanced metabolic competence, attenuated toxin‐induced alterations and produced metabolite profiles more consistent with human liver physiology, supporting the use of CYP450‐induced HepG2 models for improved *in vitro* hepatotoxicity assessment. Created in BioRender. Jinks, M. (2026) https://BioRender.com/qo8xbp5.

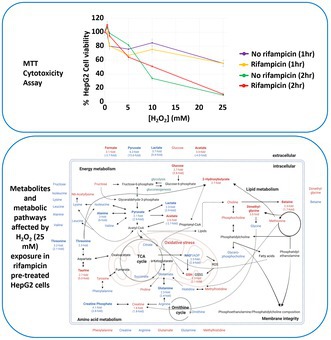

**Abbreviations**
ATP: adenosine triphosphate
AUROC: area under the receiver operator curve
BBI: broadband decoupling inverse
CO_2_
: carbon dioxide
CYP3A4: cytochrome P450 3A4
CYP450: cytochrome P450
DILI: drug‐induced liver injury
DMEM: Dulbecco's Modified Eagle Medium
DMG: dimethylglycine
DMSO: Dimethyl sulfoxide
EDTA: Ethylenediaminetetraacetic acid
EtOH: ethanol
FBS: fetal bovine serum
GPx: glutathione peroxidase
GSH: glutathione
H_2_O_2_
: hydrogen peroxide
HepG2: human liver carcinoma IVDr, *In Vitro* Diagnostics research
MeOH: methanol
MS: mass spectrometry
MTT: thiazolyl blue tetrazolium bromide
NMR: Nuclear Magnetic Resonance
OPLS‐DA: orthogonal projection to latent structures discriminant analysis
PBS: phosphate‐buffered saline
PC: principal component
PCA: principal component analysis
PCR: polymerase chain reaction
PQN: probabilistic quotient normalisation
R2X: model interpretation rate
ROS: reactive oxygen species
rpm: revolutions per minute
SE: standard error
SF‐DMEM: serum free Dulbecco's Modified Eagle Medium
SOD: superoxide dismutase
TSP: sodium 3‐(trimethylsilyl)propionate‐d

The liver is the primary site of the metabolism of drugs and, hence, the main target for drug‐induced toxicity. Exposure to drugs, herbal medicines or other xenobiotics can lead to an adverse reaction known as drug‐induced liver injury (DILI), a leading cause of many novel compounds failing during clinical studies and being consequently withdrawn [1]. The primary mechanism is biotransformation of drugs into potentially reactive compounds causing hepatotoxicity, then DILI [2]. The resulting compounds can bind to cellular macromolecules, triggering oxidative stress and mitochondrial dysfunction, and ultimately culminating in apoptotic or necrotic cell death [3]. To counteract oxidative effects, hepatocytes have a range of critical antioxidant mechanisms, including the antioxidant glutathione (GSH), the enzymes superoxide dismutase (SOD), catalase and glutathione peroxidase (GPx). However, in DILI, antioxidant capacity is disrupted, leading to a build‐up of oxidants, which triggers oxidative stress [4].

Assessment of metabolism mediated hepatotoxicity is vital in the early stages of drug development and toxicity screening because drug metabolites generated in the liver can cause unexpected liver injury, making early detection essential to prevent late‐stage failures, reduce costs and ensure patient safety. The use of *in vitro* systems that accurately mimic human *in vivo* toxicity is needed to lower compound attrition and improve drug safety [5]. The human hepatoblastoma cell line HepG2 is a valuable *in vitro* system for hepatic toxicology testing, as it has preserved the activities of some critical metabolising liver enzymes and can achieve robust and reproducible experiments [6, 7]. A major limitation of HepG2 cells is their low cytochrome P450 (CYP450) enzymatic competence, largely attributable to reduced basal expression of key drug‐metabolising enzymes under standard culture conditions, which restricts their capacity to accurately metabolise drug‐like compounds and thereby limits their utility in metabolism‐dependent toxicity assessment. This limitation can be overcome, such as by adenoviral transfection [8] or rifampicin treatment [9, 10], which strongly increases transcription of CYP450 genes and induces metabolism. In humans, the superfamily of CYP450 enzymes comprises 57 genes that encode essential membrane‐bound proteins responsible for most phase I drug metabolism reactions, mainly in the liver. The CYP1, 2 and 3 protein families of CYP450 are implicated in the breakdown of more than 80% of all prescription drugs [11], of which CYP3A4 breaks down 50% [12]. CYP450 enzymes use iron to oxidise compounds, producing and releasing H_2_O_2_ [13, 14]. Inhibition of CYP450 enzymes may lead to toxicity or a lack of drug efficacy [15].

Metabolomics provides a powerful approach for investigating cellular responses to toxic insults, with proton nuclear magnetic resonance (^1^H NMR) spectroscopy and mass spectrometry (MS) being the two principal analytical platforms. ^1^H NMR was selected in this study because it provides highly reproducible, nondestructive and inherently quantitative measurements with minimal sample preparation, making it well‐suited for comparative assessment of toxin‐induced metabolic changes. In contrast, MS generally offers greater sensitivity and broader metabolite coverage, allowing detection of many low‐abundance metabolites that may not be observable by NMR. Therefore, while the present approach enables reliable quantification of major intra‐ and extracellular metabolites, some oxidative stress‐associated compounds, particularly short‐lived reactive species and low concentration antioxidant intermediates, may not have been detected. Nevertheless, ^1^H NMR provides robust coverage of central carbon metabolism, amino acid metabolism, energy metabolism and pathways associated with cellular redox balance, making it an appropriate platform for evaluating global metabolic responses to oxidative stress and hepatotoxicity.

Since DILI is influenced by both daily toxin dose and liver injury, we investigated the metabolic effects of low, medium and high concentrations of the oxidative stress‐inducing model toxin H_2_O_2_ in HepG2 cells to assess whether ^1^H NMR spectroscopy could detect toxin‐induced metabolomic changes across a range of concentrations. Furthermore, the metabolic effects of rifampicin pretreatment to induce CYP450 enzymes in HepG2 cells were examined by comparing the intra‐ and extracellular metabolite profiles of cells exposed to 25 mm H_2_O_2_. This allows the model to more accurately mimic human liver metabolism and detect toxicity mediated by metabolites, improving the relevance of *in vitro* hepatotoxicity studies.

## Materials and methods

### Cell culture

The adherent human hepatoma cell line HepG2 was obtained from the European Collection of Authenticated Cell Cultures (Salisbury, United Kingdom) (HepG2 cell accession number: CVCL_0027) and provided by Dr Ian Musgrave (University of Adelaide, South Australia). All cell lines used in this work are authenticated at least every 3 years using STR profiling following the protocol recommended by ATCC. Profiling data were compared against the ATCC STR database using a cut‐off of > 80%. All experiments were performed with mycoplasma‐free cells, confirmed by PCR. Cells were maintained in Dulbecco's Modified Eagle Medium (DMEM) supplemented with 1% nonessential amino acids, 1% penicillin/streptomycin and 10% heat‐inactivated fetal bovine serum (FBS). Cultures were grown as monolayers in 75 cm^2^ flasks at 37°C in a humidified 5% CO_2_ atmosphere, with medium replaced every 24–48 h until 80%–90% confluency, after which cells were passaged or used experimentally.

For subculturing, cells were washed with 5 mL pre‐warmed phosphate‐buffered saline (PBS) and detached with 2 mL of 0.25% trypsin–EDTA. Trypsin was neutralised with 8 mL prewarmed DMEM, and cells were split to a 1:5 ratio and placed into 75 cm^2^ flasks containing fresh medium [16]. Passages 17–20 and 43–45 were used for experiments.

### Experimental design

Rifampicin (10 mm in DMSO; Merck, Castle Hill, NSW, Australia) was diluted 1:5 in PBS, which was then further diluted with DMEM containing 10% FBS. For cells pretreated with rifampicin, final exposure concentrations were 18 μM rifampicin and 0.18% DMSO. Hydrogen peroxide (H_2_O_2_, 30% w/v; Rowe Scientific, Wangara, WA, Australia) was diluted in serum‐free DMEM (SF‐DMEM) to final concentrations of 0.5, 1.0, 5, 10 and 25 mm.

Cell viability was assessed using the 3‐(4,5‐dimethylthiazol‐2‐yl)‐2,5‐diphenyltetrazolium bromide (MTT) assay as described by Siddiqui [17]. In brief, HepG2 cells were seeded at a density of ~1.2 × 10^4^ cells/well in 96‐well culture plates for 48 h at 5% CO_2_ and 37°C to allow for adhesion. Half of the cells were incubated with supplemented DMEM (200 μL/well) while the other half received 100 μL of DMEM + rifampicin and 100 μL supplemented DMEM treatment/well to stimulate phase I metabolic activity of CYP450 enzymes. After 1 or 2 h exposure to 0.5, 1, 5, 10 or 25 mm H_2_O_2_, medium was aspirated, and MTT solution (0.25 mg/mL MTT in SF‐DMEM) was added (100 μL/well), and plates incubated for 3 h. Viable cells metabolise MTT into formazan product with a maximum absorbance of 570 nm. Non‐viable cells cannot convert MTT into formazan; hence, colour development is a marker of cell viability [18]. After 3 h incubation, the MTT solution was discarded, and 100 μL of DMSO was added to each well and mixed gently until the cells had lysed and the dye was dissolved uniformly. Absorbance was read at 570 nm using a Tecan Spark microplate reader. Untreated controls were run under identical conditions. Each experimental group consisted of 15 replicates.

For experimental purposes, cells were seeded in six‐well tissue culture plates at a density of ~2.0 × 10^6^ cells using 1 mL of DMEM containing Rifampicin and 1 mL of supplemented DMEM per well. Plates were incubated at 37 °C in a humidified incubator with 5% CO_2_ for 48 h, allowing cell adhesion prior to exposure to treatment with hydrogen peroxide at final concentrations of 0 mm, 1 mm, 10 mm and 25 mm H_2_O_2_ for 2 h. Each treatment condition was conducted using a separate six‐well plate, providing five biological replicates per group. One well per plate was reserved for determining viable cell numbers for subsequent normalisation of metabolomic datasets.

Cell counts were assessed using the Trypan blue exclusion method [19]. DMEM was removed, and cells were rinsed twice with 1 mL PBS to eliminate all extracellular remnants. Cells were then detached by adding 500 μL of 0.25% trypsin–EDTA per well and incubating for 5 min. Trypsin activity was quenched with 1.5 mL DMEM per well. A 100 μL aliquot of the resulting cell suspension was combined 1:1 with 0.4% Trypan blue, and viable cells were manually enumerated using 10 μL of the mixture on an improved Neubauer haemocytometer under an Olympus CKX41 inverted light microscope.

### Sampling of intracellular metabolites

Following 2 h of toxin treatment, six‐well plates were removed from the incubator and immediately placed on ice. Metabolic activity was quenched by aspirating the culture medium and rinsing each well with 1 mL of ice‐cold PBS. After removal of PBS, 250 μL of methanol (MeOH) was added to each well, and cells were detached by manually scraping the well surface into the solvent. The cell scraper was cleaned with 70% ethanol (EtOH) followed by distilled water between samples.

Intracellular metabolites were extracted by disrupting cell membranes and separating metabolites from cellular debris using a tissue lyser operating at 6500 rpm for 2 × 20 s cycles [16]. Samples were subsequently centrifuged at 16 100 × ***g*** for 1 min at 4 °C to pellet residual cellular material. A volume of 300 μL of the resulting supernatant was transferred to new microcentrifuge tubes and dried using a speed vacuum concentrator. Dried extracts were stored at −80 °C until further analysis.

### Sampling of extracellular metabolites

For the assessment of extracellular metabolites, the spent culture medium was removed from the six‐well plates and centrifuged at 13 000 × ***g*** for 10 min at 4 °C. The resulting supernatant samples were transferred into fresh microcentrifuge tubes and stored at −80 °C prior to analysis.

### ^1^H NMR spectroscopy analysis

#### Sample analysis of cell extracts

Cell extracts were reconstituted in 540 μL D_2_O, followed by the addition of 60 μL of buffer (1.5 m KH_2_PO_4_, 2 mm NaN_3_, 0.1% sodium 3‐(trimethylsilyl)propionate‐d4 (TSP), pH 7.4). A total volume of 600 μL was transferred into a Bruker SampleJet NMR tube, which were sealed and mixed by gentle inversion.

All NMR spectra were acquired on a Bruker 600 MHz Bruker Avance III HD spectrometer at 300 K and equipped with a BBI probe and a SampleJet robotic cooling system maintained at 5 °C. Quantitative calibration of the instrument was performed prior to sample acquisition in accordance with established protocols [20]. Each sample was analysed using a single automated run with a total acquisition time of 29 min, employing a one‐dimensional ^1^H experiment with solvent suppression (pulse program: noesygppr1d, 256 scans, 65 536 data points, spectral width of 12019.23 Hz). Data were processed in automation using Bruker Topspin version 3.6.4 and ICON™ NMR to achieve phasing and baseline correction and spectra were referenced to TSP at δ 0.00.

#### Sample analysis of cell supernatants

Samples were centrifuged at 13 000 × ***g*** for 10 min at 4 °C [21]. A volume of 540 μL of the resulting supernatant was mixed with 60 μL of buffer (1.5 M KH_2_PO_4_, 2 mm NaN_3_, 0.1% TSP, pH 7.4) and transferred into standard 5 mm NMR tubes. Proton NMR spectra were acquired using a ^1^H 1D experiment with solvent presaturation, with each acquisition duration 4 min (pulse program: noesygppr1d, 32 scans, 65 536 data points, spectral width 12019.23 Hz). Data were processed in automation using Bruker Topspin version 3.6.4 and ICON™ NMR to achieve phasing and baseline correction, and spectra were referenced to TSP at δ 0.00.

### Data processing and statistical analysis

All multivariate analyses were performed using the *metabom8* package (version 0.4.4, https://tkimhofer.github.io/metabom8/). Spectral regions corresponding to the water signal (δ 4.66–5.16) and noise (δ < −1, and δ >13) were excluded. Data were normalised using probabilistic quotient normalisation (PQN) prior to multivariate analysis, which included principal component analyses (PCA) and orthogonal partial least squares discriminant analysis (O‐PLS‐DA).

Relative metabolite concentrations in HepG2 cell extracts exposed to varying H_2_O_2_ concentrations were determined by integrating relevant spectral regions. For supernatant samples, absolute metabolite concentrations (mm) were calculated using the IVDr quantification reference (10 mm) at 12 ppm. Fold changes and paired *t*‐tests were performed, and log_2_ fold changes greater than 1 or less than −1 with *P*‐values < 0.05 were considered statistically significant between control and H_2_O_2_‐treated groups. Comparisons of log_2_ fold changes and associated *P*‐values were made across low, medium and high H_2_O_2_ treatments with nontreated cells. The comparison of 25 mm H_2_O_2_‐treated cells with untreated cells was previously reported, and the results from that study are incorporated here [22].

## Results

### Determination of H_2_O_2_ exposure time and concentration

**Fig. 1:**
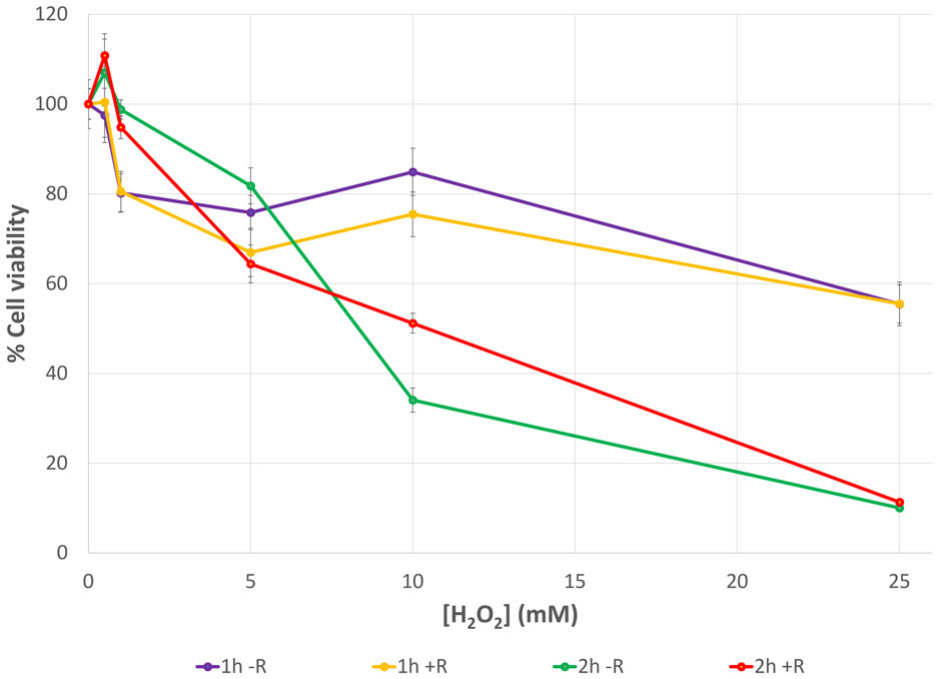
Changes in HepG2 cell viability (%) when pre‐incubated with rifampicin (+R) following 1 h (orange) and 2 h (red) exposure and when not treated with rifampicin (−R) following 1 h (purple) and 2 h (green) exposure to 0 mm, 0.5 mm, 1, 5 mm, 10 mm and 25 mm H_2_O_2_ as determined by an MTT (thiazolyl blue tetrazolium bromide) assay (n = 15 for each concentration). Cell viability was directly proportional to absorbance at 570 nm. Data are expressed as the mean ± SE (Standard Error).

The MTT assay showed that H_2_O_2_ inhibited HepG2 cell viability in a concentration‐ and time‐dependent manner, with broadly similar responses observed in both rifampicin pretreated (+R) and non‐pretreated (−R) cells. After 1 h exposure, cell viability remained close to control levels at low concentrations (0.5–1 mm) but decreased progressively with increasing H_2_O_2_ concentration, reaching approximately 55% at 25 mm in both +R and −R conditions. A more pronounced concentration‐dependent reduction in viability was observed after a 2 h exposure. In +R‐treated cells, viability decreased to 11% ± 0.45 at 25 mm H_2_O_2_. A similar pattern was observed in ‐R cells, where viability declined to 10% ± 0.48 at 25 mm exposure (Fig. 1). Low (1 mm), medium (10 mM) and high (25 mm) concentrations of H_2_O_2_, with a 2 h exposure time, were used for the subsequent experiments in this study.

### ^1^H NMR analysis of HepG2 cell extracts, and supernatants

33 and 26 metabolites were assigned in the spectra of cell extracts and supernatants, respectively. Acetate, alanine, betaine, creatine, formate, glucose, glutamine, glutathione, lactate and threonine dominated the ^1^H NMR spectra of HepG2 cell extracts. In the ^1^H NMR spectra of HepG2 cell supernatants, key metabolites included acetate, alanine, ethanol, formate, glucose, lactate, methionine, pyruvate, taurine, threonine and tyrosine (Table S1, Fig. S1).

### Dose‐dependent effects of toxin exposure on metabolite profiles

**Fig. 2:**
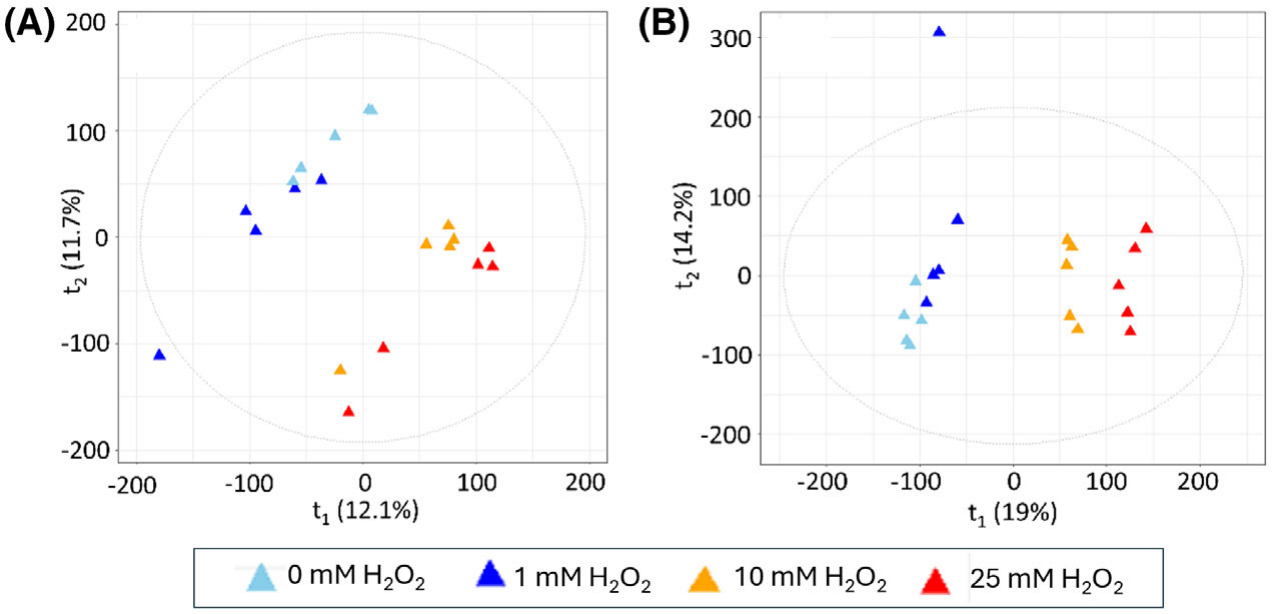
Principal component analysis scores plots showing the (A) intracellular metabolic effects and (B) extracellular metabolic effects on HepG2 cells of no treatment (light blue), low (1 mm) (blue), medium (10 mm) (orange) and high (25 mM) (red) H_2_O_2_ concentrations (n = 5 per group).

PCA of the NMR spectral data from cell extracts and supernatants revealed a clear separation of metabolite profiles from the different H_2_O_2_ treatment groups, although untreated and 1 mm H_2_O_2_ were clustered. A similar trend was observed with 10 mm and 25 mm treatments. The first two principal components (PC1 and PC2) explained 12.1% and 11.7% of the variance in the HepG2 cell extracts and 19% and 14.2% for the HepG2 cell supernatants. 95% of the samples were distributed within the 95% confidence interval (Fig. 2).

Metabolite concentrations in treated HepG2 cells followed similar trends of increase or decrease with rising toxin concentration compared to controls. Significant fold changes in cells with low toxin exposure (1 mm) were observed for four metabolites (acetate, alanine, arginine and glutamate), all significantly increasing. Twelve metabolites were significantly different with 10 mm exposure, while 20 metabolites were significantly different with 25 mm exposure. We observed reductions in alanine (1.35‐ to 7.80‐fold), lactate (1.49‐ to 3.92‐fold decrease), NAD^+^ (1.37‐ to 2.27‐fold), and pyruvate (1.45‐ to 2.70‐fold) with 10 mm and 25 mm H_2_O_2_ treatment, respectively. The levels of glutathione (2.04‐ to 2.07‐fold) were significantly increased with 10 mm and 25 mm toxin treatment, as shown in Supplementary Table 1.

In the corresponding cell supernatant samples, significant alterations in metabolite concentrations were observed following toxin exposure. Acetate levels increased in a concentration‐dependent manner at 1 mm, 10 mm, and 25 mm, while formate increased at the low and medium concentrations but significantly reduced at the high concentration. In contrast, lactate, pyruvate and threonine levels decreased across all exposure concentrations, with progressively greater reductions observed at higher toxin levels (Table S2).

### Cellular metabolic responses to rifampicin pretreatment

**Fig. 3:**
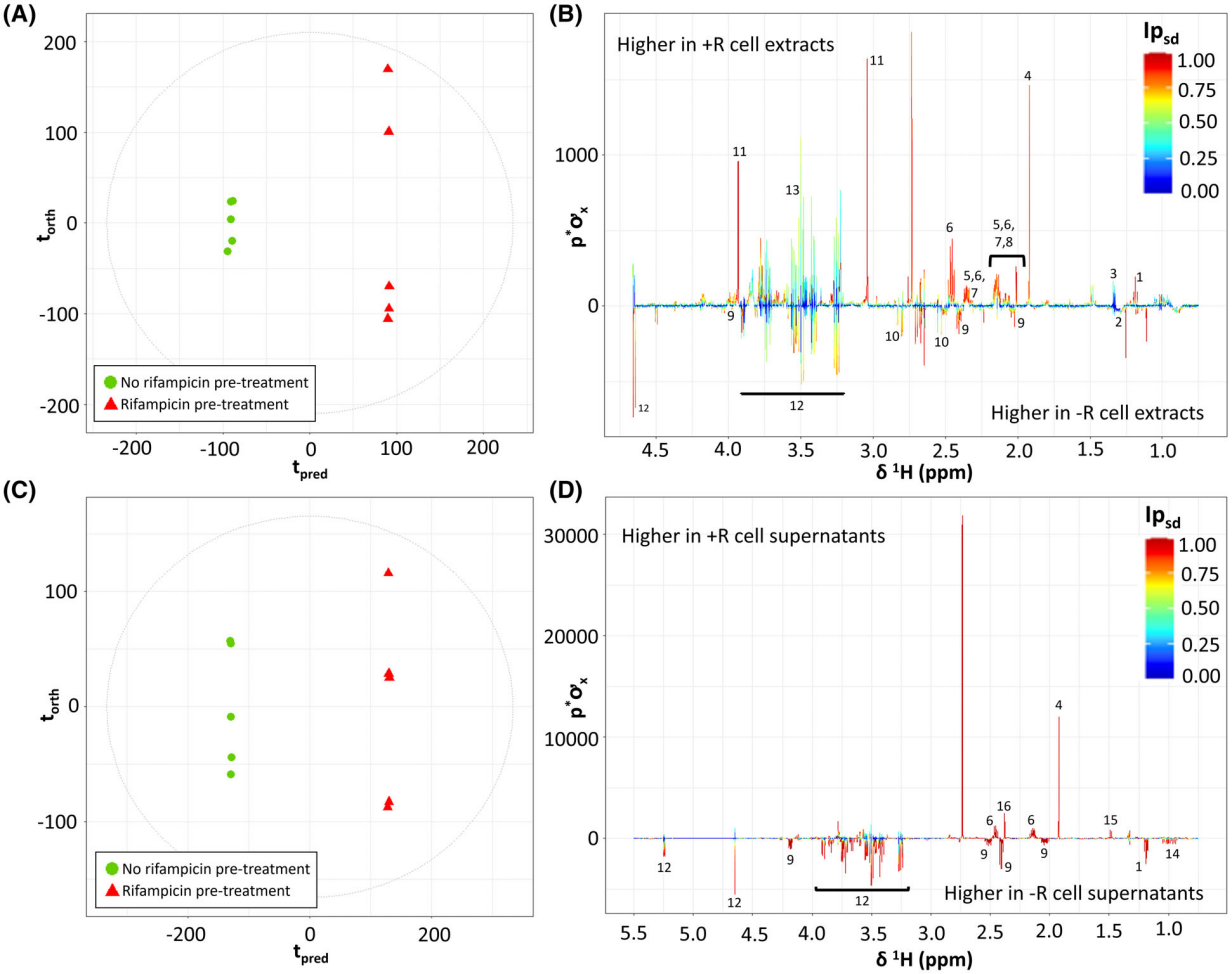
Orthogonal projection to latent structures discriminant analysis scores plots area under the receiver operator curve = 1.0 and corresponding coefficient loadings plots derived from ^1^H NMR spectra of HepG2 cell extracts (A, B) and supernatants (C, D) with (red) and without (green) rifampicin pretreatment before exposure to 25 mm H_2_O_2_ for 2 h (n = 5/ group). (1) ethanol, (2) lactate, (3) threonine, (4) acetate, (5) glutamate, (6) glutamine, (7) glutathione, (8) methionine, (9) pyroglutamate, (10) citrate, (11) creatine, (12) glucose, (13) glycine, (14) branched‐chained amino acids, (15) alanine, (16) pyruvate.

O‐PLS‐DA from the NMR spectral data of HepG2 cells and their corresponding supernatants revealed a clear separation of metabolite profiles between those that were pretreated with rifampicin and those that were not, with 100% of both cell extract and supernatant samples located within the 95% confidence interval in the O‐PLS‐DA scores plots. Parameters for the classification of +R versus ‐R groups for the cell extracts and supernatants were respectively R^2^X = 20% and 39%, while the area under the AUROC and CV AUROC were 1.0 for both O‐PLS‐DA models, which verified a satisfactory goodness of fit and excellent predictability (Fig. 3A,C). In the corresponding O‐PLS‐DA coefficient loading plots, significant biochemical distinctions between the +R and ‐R groups were identified, namely acetate, creatine, glutamate, glutamine, glutathione and methionine were substantially higher in the +R cell extract profiles, whereas citrate, glucose and pyroglutamate were identified to be significantly higher in the ‐R cell extract group. In the corresponding +R supernatant profiles, acetate, alanine, glutamine and pyruvate were determined to be higher. In contrast, branched‐chain amino acids, glucose, pyroglutamate and lactate were higher in the ‐R supernatant group (Fig. 3B,D).

Overall, fold changes in metabolites from the ‐R groups were substantially greater than +R groups. For example, glucose increased 2.6‐fold in the +R and 3.7‐fold in the ‐R cell extract samples, and pyruvate decreased 8.5‐fold in the +R and 223‐fold in the ‐R cell supernatant samples. Notable differences were observed in phosphocholine levels, which rose in +R but declined in ‐R cell extracts. *N,N*‐dimethylglycine and N6‐acetyllysine concentrations substantially increased by threefold and 3.3‐fold in the +R extract group, whereas no change could be seen for these metabolites in the ‐R group. 2‐hydroxybutyrate (↑2.7‐fold), NAD^+^ (↓2.5‐fold), and threonine (↓2.5‐fold) levels changed substantially more in the +R versus the ‐R extract samples (Supplementary Table 3).

## Discussion

**Fig. 4:**
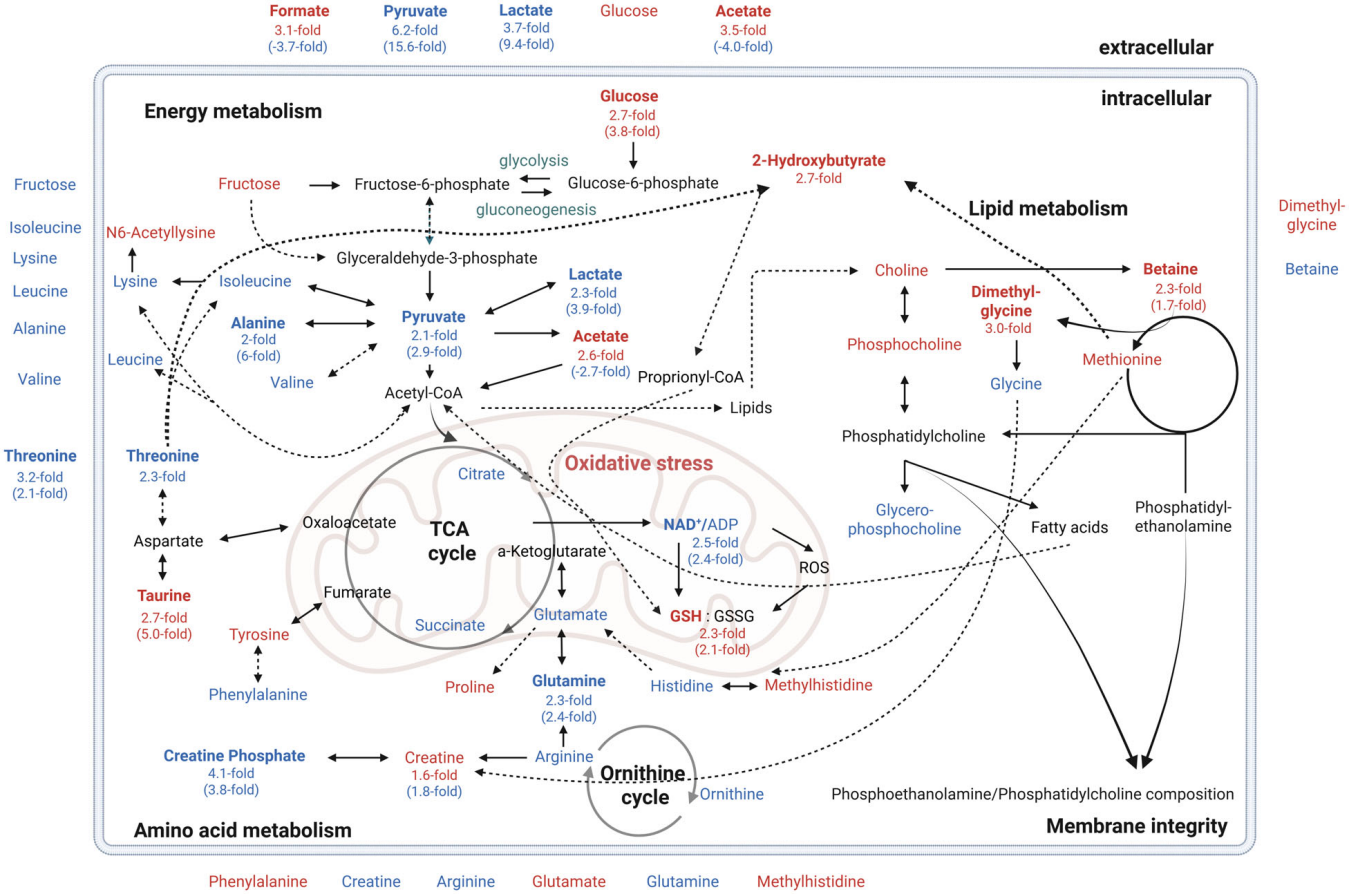
Metabolites and metabolic pathways affected by H_2_O_2_ (25 mm) exposure in rifampicin pretreated HepG2 cells as detected by ^1^H NMR‐based metabolomics. Metabolites in bold indicate significant fold increases (red) and decreases (blue) (fold change of >2 or <0.5) in metabolite concentrations compared to controls. Fold changes in brackets show those from without rifampicin pretreatment, in which CYP450 metabolism was not induced in HepG2 cells before toxin exposure. Created in BioRender. Jinks, M. (2026) https://BioRender.com/qo8xbp5.

In this study, we demonstrate that ^1^H NMR spectroscopy can detect metabolomic changes resulting from exposure to varying concentrations of H_2_O_2_. Several key metabolites and major metabolic pathways were altered by H_2_O_2_ exposure, as summarised in Fig. 4. Interestingly, the intra‐ and extracellular metabolite profiles of the 1 mm H_2_O_2_ group largely clustered with those of the controls, although several metabolites exhibited significant fold changes. In contrast, the metabolite profiles from the medium H_2_O_2_ concentration (10 mm) samples clustered with those of the high toxin exposure group (25 mm). It should be noted that at 25 mm H_2_O_2_ cell viability becomes markedly reduced. Consequently, the observed metabolic signatures likely reflect a combination of regulated cell responses but also some non‐specific effects associated with cell death. These include loss of membrane integrity, resulting in the passive release of intracellular metabolites into the extracellular environment. However, these findings demonstrate that NMR spectroscopy enables the determination of the lowest toxin concentration capable of eliciting measurable metabolic perturbations, providing an efficient and cost‐effective alternative to conventional animal models.

Furthermore, we demonstrated that CYP450 enzyme activity has a metabolic effect in HepG2 cells exposed to H_2_O_2_. The 25 mm H_2_O_2_ concentration was selected for the rifampicin comparison as it produced the most pronounced metabolomic alterations, thereby providing the greatest analytical sensitivity for detecting CYP450 dependent modulation of the metabolic responses. While this concentration induces substantial cytotoxicity and reduced cell viability, it was specifically chosen to ensure a strong oxidative stress challenge, enabling evaluation of whether rifampicin induced CYP450 activity could modify the metabolic profile under conditions of maximal metabolic disturbance.

Although the metabolism of the cells was disrupted by toxin exposure, metabolic responses in cells pretreated with rifampicin 48 h before toxin exposure differed, particularly in their metabolic compensation mechanisms to counteract oxidative stress induced by H_2_O_2_. Overall, fold changes in intracellular metabolite concentrations were less pronounced in rifampicin pretreated cells compared to untreated cells. Notably, metabolite exchange with the extracellular medium was minimally affected by toxin exposure in pretreated cells, suggesting an altered metabolic stress response and improved maintenance of metabolic homeostasis under oxidative conditions.

In comparison, significant fold changes in the extracellular concentrations of numerous metabolites were observed in cells without CYP450 induction. This indicates that rifampicin pretreatment alters metabolic responsiveness under oxidative stress conditions, likely due to rifampicin‐induced transcriptional and metabolic reprogramming. [14]. While CYP450 enzymes are associated with xenobiotic metabolism and endogenous lipid pathways, their alterations here should be interpreted here as a marker of altered hepatic metabolic state. Notably, extracellular acetate levels substantially increased with rifampicin pretreatment, whereas they significantly decreased in non‐pretreated cells. Extracellular lactate, pyruvate and threonine concentrations significantly decreased with and without CYP450 induction, suggesting attenuation of stress‐induced metabolic disruption rather than activation of distinct metabolic pathways.

Overall, the metabolic stress responses observed in cells pretreated with rifampicin in this study reflect a metabolically reprogrammed hepatocyte like state with altered stress responsiveness. While rifampicin is known to induce cytochrome P450 expression *in vitro* in hepatocyte models [23], in the present context of direct oxidative stress (H_2_O_2_ exposure), this does not translate into CYP450‐mediated detoxification. Instead, it likely reflects altered baseline redox buffering and metabolic adaptation capacity.

### Energy metabolism

Exposure to high concentrations of H_2_O_2_ caused pronounced disruption of energy metabolism in HepG2 cells. In rifampicin pretreated cells exposed to 25 mm H_2_O_2_, intracellular glucose levels increased significantly, mirroring the response observed in non‐pretreated cells; however, rifampicin pretreatment did not alter glucose uptake from the extracellular medium relative to untreated controls. This pattern suggests a reduction in intracellular ADP generation, resulting in glucose accumulation within the cell. Consistent with this interpretation, intracellular lactate, oxidised NAD^+^ and pyruvate levels were significantly decreased [24]. Notably, although ADP, glucose, lactate and pyruvate concentrations were significantly perturbed in rifampicin‐treated cells, the magnitude of these changes was less severe than in non‐pretreated cells, implying partial metabolic compensation through alternative ATP‐generating pathways, such as the phosphocreatine system, in response to impaired glycolytic flux.

Furthermore, phosphocholine levels were elevated in pretreated cells compared with the markedly reduced levels observed in non‐pretreated cells, which may reflect preserved ATP availability to support choline phosphorylation; however, this increase could also plausibly arise from enhanced membrane phosphatidylcholine turnover under oxidative stress, where reactive oxygen species promote phospholipid breakdown and subsequent recycling, leading to phosphocholine accumulation [25]. We also observed that rifampicin pretreatment affected intra‐ and extracellular acetate concentrations. Acetate significantly increased, suggesting increased *de novo* generation of intracellular acetate from intra‐ and extracellular pyruvate [26] in response to cellular energy depletion to ultimately increase intracellular acetyl‐CoA pools, which can then be used to supply cellular energy [27, 28].

### Amino acid metabolism and oxidative stress

This study revealed significant perturbations in the metabolism of several amino acids with antioxidant properties following H_2_O_2_ treatment. Here, we found intracellular levels of several amino acids to be affected, namely betaine, *N,N*‐dimethylglycine (DMG), glutamine, glutathione, taurine and threonine. The marked depletion of intracellular glutamine alongside elevated glutathione levels suggests that glutamine was actively catabolised to support glutathione biosynthesis under oxidative stress [29] to detoxify reactive oxygen species (ROS) such as H_2_O_2_ using catalase and GPx. Consistent with Gall et al. oxidative stress or drug detoxification in the liver can markedly increase the rate of hepatic glutathione synthesis in humans. In this study, levels of 2‐hydroxybutyrate were also significantly increased but not detected in cells without rifampicin pretreatment [30]. This organic acid is generated from alpha‐ketobutyrate, which is derived from the catabolism of threonine and methionine and glutathione synthesis. 2‐hydroxybutyrate is metabolised to propionyl‐CoA and carbon dioxide. Shifts in glutathione synthesis have been shown to correlate with increased production of 2‐hydroxybutyrate through cysteine metabolism, reflecting altered glucose handling and enhanced oxidative stress associated with lipid peroxidation [30, 31].

Numerous studies have shown that methionine plays a vital role in the oxidative stress response, scavenging ROS [32]. In this study, intra‐ and extracellular methionine levels were increased and unaffected, respectively. In comparison, methionine concentration was unchanged within HepG2 cells and significantly increased in the extracellular medium without rifampicin pretreatment. This suggests that ‐R cells could not take up extracellular methionine to provide antioxidant capacity; hence, metabolism was reduced. Further, intra‐ and extracellular pyruvate levels were notably reduced in HepG2 cells with and without rifampicin pretreatment, though not to the same extent as previously seen. Pyruvate has antioxidant properties that reduce mitochondrial ROS production [33], and its significant depletion indicates the cells are in a state of cellular oxidative stress after H_2_O_2_ exposure.

### Lipid metabolism

Kamphorst *et al*. [24] revealed that acetate is vital to maintain lipid biosynthesis when the availability of glucose and glutamine‐derived carbon is diminished, as observed in both of our studies. Higher intracellular acetate levels in rifampicin‐treated cells suggest increased generation of lipogenic acetyl‐CoA in response to oxidative stress. Furthermore, an increase of intracellular N6‐acetyllysine was detected, indicating activation of the lysine degradation pathway to stimulate biosynthesis of acetoacetyl‐CoA, an essential precursor for the generation of cholesterol [34]. Increased intracellular betaine, choline, DMG, methionine, phosphocholine and decreased glycerophosphocholine levels in rifampicin‐treated cells support these findings, as these metabolites are crucial for the generation of phospholipids [35], and reflect an adaptive cellular stress response to maintain membrane integrity and boost antioxidant levels to counteract lipid peroxidation induced by oxidative stress [36, 37]. Nonetheless, although cells pretreated with rifampicin appear to have a higher metabolic capacity to respond to cellular stress, levels of oxidative damage seem to have overwhelmed repair capacity in cells treated with 25 mm H_2_O_2_, regardless of rifampicin treatment.

A limitation of this study is the absence of a vehicle control for rifampicin treatment. As rifampicin was dissolved in DMSO, potential solvent‐related metabolic effects cannot be completely excluded. Future studies should include a matched DMSO control to distinguish the effects of CYP450 induction from those associated with solvent exposure. In future studies, it would be interesting to compare the metabolism and antioxidant capacity of HepG2 cells with and without rifampicin treatment postexposure to known xenobiotics requiring CYP450 bioactivation, such as paracetamol [38].

## Conclusions

This study demonstrates that ^1^H NMR spectroscopy is a sensitive and effective approach for characterising metabolomic alterations in HepG2 cells exposed to varying concentrations of H_2_O_2_. Distinct concentration‐dependent metabolic responses were observed, with low‐dose exposure (1 mM) producing minimal global perturbations, while higher concentrations (10–25 mm) induced pronounced and overlapping metabolomic profiles associated with severe oxidative stress and reduced cell viability. At the highest concentration, metabolic signatures likely reflected both regulated stress responses and nonspecific effects of membrane damage and cell death, highlighting the importance of dose consideration when interpreting toxicity related metabolomic data.

Importantly, rifampicin‐mediated induction of CYP450 enzymes significantly modified the cellular metabolic response to oxidative stress. Pretreated cells exhibited attenuated fold changes in key intracellular metabolites and reduced disruption of extracellular metabolite exchange, suggesting enhanced metabolic resilience and partial maintenance of homeostatic control under oxidative conditions. These findings indicate that CYP450 induction is associated with a reprogrammed hepatocyte‐like metabolic state, characterised by altered energy metabolism, amino acid turnover and lipid‐associated pathways.

Overall, the results support the utility of NMR‐based metabolomics as a rapid and cost effective platform for detecting toxin‐induced metabolic perturbations and for evaluating modulatory effects of hepatic enzyme induction. This approach provides valuable insight into oxidative stress responses and highlights the potential of metabolomic profiling for improving *in vitro* models of liver toxicity and xenobiotic metabolism.

## Conflict of interest

The authors declare no conflicts of interest.

## Author contributions

GLM, BAB and SL conceived and designed the project. GLM and ECD contributed to methodology development for cell work. SLL contributed to methodology development for NMR analysis, curated the NMR data. MJ and ECD performed the experiments. MJ, GLM, ECD and SL analysed and interpreted the data. GLM, BAB and SL supervised the study. MJ wrote the original manuscript. All authors read, edited and approved the manuscript.

## Supporting information


**Fig. S1.** Overlaid NMR spectra of extracellular metabolic effects on HepG2 cells with and without H_2_O_2_ treatment.
**Table S1.** Quantitative comparison of the metabolites found in HepG2 cell extracts treated with H_2_O_2_.
**Table S2.** Quantitative comparison of the metabolites identified in HepG2 cell supernatants treated with H_2_O_2_.
**Table S3.** Quantitative comparison of metabolites found in rifampicin (+R) treated versus untreated (−R) HepG2 cell extracts and supernatants exposed to H_2_O_2_.

## Data Availability

The data that support the findings of this publicly available and can be found here: https://doi.org/10.5281/zenodo.20725893.
