# Supplementary material for: Dose‐dependent hepatotoxicity of hydrogen peroxide in HepG2 cells and its modulation by CYP450 induction
Source: FEBS Open Bio. 2026 Jun 28:10.1002/2211-5463.70299. Online ahead of print. doi: 10.1002/2211-5463.70299 (PMC13398862; doi:10.1002/2211-5463.70299)
Supplement: Supplementary file 1 — Fig. S1. Overlaid NMR spectra of extracellular metabolic effects on HepG2 cells with and without H2O2 treatment. Table S1. Quantitative comparison of the metabolites found in HepG2 cell extracts treated with H2O2. Table S2. Quantitative comparison of the metabolites identified in HepG2 cell supernatants treated with H2O2. Table S3. Quantitative comparison of metabolites found in rifampicin (+R) treated versus untreated (−R) HepG2 cell extracts and supernatants exposed to H2O2. [file FEB4-9999-0-s001.pdf]

Dose Dependent Hepatotoxicity of Hydrogen Peroxide in HepG2 Cells and Its Modulation by CYP450 Induction: A ^{1}H NMR Metabolomics Study

Maren Jinks[1,2,3,4,5], Garth L. Maker[1,2], Emily C. Davies[1,2], Berin A. Boughton[6], Samantha Lodge[1*]

[1]Centre for Computational and Systems Medicine, Health Futures Institute, Harry Perkins Building, Murdoch University, Perth, WA 6150, Australia

[2]Medical, Molecular and Forensic Sciences, Murdoch University, 90 South Street, Murdoch, WA 6150, Australia

[3]Leukaemia Translational Research Laboratory, WA Kids Cancer Centre, The Kids Research Institute Australia, Perth, WA 6009, Australia

[4]Curtin Medical School, Curtin University, Perth, WA 6102, Australia

[5]Department of Microbiology, PathWest Laboratory Medicine, Perth, WA 6009, Australia

[6]La Trobe Institute for Sustainable Agriculture and Food, AgriBio, La Trobe University, Bundoora, VIC 3083, Australia

*Corresponding author at: Murdoch University, 90 South Street, Murdoch, WA, Australia 6150. Samantha Lodge: sam.lodge@murdoch.edu.au

Keywords: HepG2, hydrogen peroxide, *in vitro* toxicology, model systems, cytochrome P450, metabolomics, NMR spectroscopy.

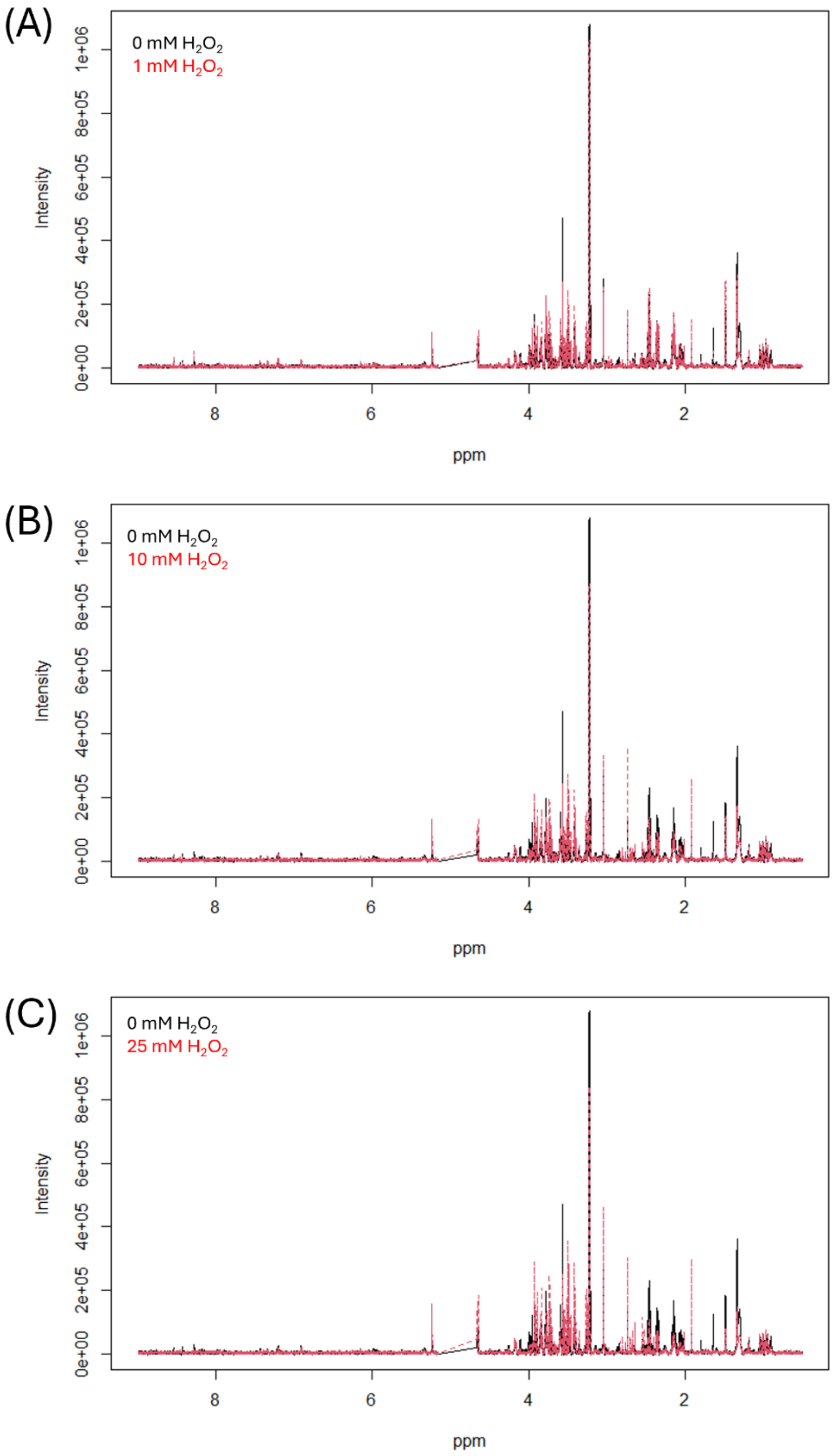

Figure S1 – Overlaid NMR spectra of extracellular metabolic effects on HepG2 cells with (A) no treatment (black) and those treated with 1 mM H_2O_2, (B) no treatment (black) and those treated with 10 mM H_2O_2 and (C) no treatment (black) and those treated with 25 mM H_2O_2.

[OFFICIAL]

Supplementary Table 1 – Quantitative comparison of the metabolites found in HepG2 cell extracts treated with 1 mM (n=5), 10 mM (n=5), and 25 mM H_2O_2 for 2 h (n=5). All log_2 fold changes and *p*-values are compared to control samples. Resonances in bold were used to quantify metabolite concentrations. Statistical significance (log_2 fold change > 1 or < -1) is indicated in bold ($p < 0.05$). BCAA – Branch chain amino acid.

**Metabolites**	**δ ¹H ppm and multiplicity**	**Log_2FC (1 mM)**	***p*-value (1 mM)**	**Log_2FC (10 mM)**	***p*-value (10 mM)**	**Log_2FC (25 mM)**	***p*-value (25 mM)**
acetate	**1.93 (s)**	+1.13	**0.008**	+1.00	0.12	-1.33	**<0.001**
alanine	**1.48 (d)**, 3.78 (q)	+0.35	**0.01**	-0.43	**0.01**	-2.97	**<0.001**
arginine	1.91 (m), 1.64 (m), 3.24 (t), **3.78 (t)**	+0.93	**0.003**	+0.15	0.75	-	-
ADP	**4.22 (m)**, 4.29 (m), 4.59 (t), 6.13 (d), 8.26 (s), 8.52 (s)	-0.25	0.24	-0.45	**0.04**	-1.17	**<0.001**
betaine	3.27 (s), **3.89 (s)**	+0.38	0.08	+0.30	0.21	+0.83	**<0.001**
choline	**3.19 (s)** ,4.05 (m), 3.51 (m),	+0.84	0.09	+0.63	0.17	-	-
citrate	2.53 (d), **2.64 (d)**	+0.01	0.67	-0.32	0.07	+1.12	**<0.001**
creatine	**3.04 (s)**, 3.94 (s)	+0.10	0.22	+0.67	**0.01**	-1.91	**<0.001**
formate	**8.46 (s)**	-0.10	0.87	+0.31	0.11	+1.15	**<0.001**
glucose	3.23 (dd), 3.39 (m), 3.49 (t), 3.72 (dd), 3.81 (dd), 3.88 (dd), 4.63 (d), **5.24 (d)**	+0.28	0.09	+0.55	0.19	+1.90	**<0.001**
glutamate	2.11 (m), 2.33 (m), **3.75 (m)**	+0.62	**0.02**	-0.17	0.44	-1.23	**<0.001**
glutamine	2.13 (m), **2.44 (m)**, 3.78 (m)	+0.11	0.11	-0.77	**0.01**	-1.18	**<0.001**
glutathione	2.18 (m), 2.57 (m), 2.97 (dd), 3.31 (dd), 3.77 (m), **3.94 (s)**, 4.55 (dd)	+0.12	0.22	+1.03	**0.002**	+1.05	**0.005**
glycerophosphocholine	3.23 (s), **3.69 (m)**, 3.92 (m), 4.32 (dd)	+0.07	0.12	-0.12	0.08	-	-
glycine	**3.58 (s)**	+0.10	0.07	-0.31	**0.01**	-0.18	**<0.001**
isoleucine (BCAA)	0.96 (t), **1.02 (d)**, 1.46 (m)	+0.45	0.08	-0.04	0.57	-0.13	0.056
lactate	**1.32 (d)**, 4.12 (q)	-0.13	0.34	-0.57	**0.04**	-1.97	**<0.001**
leucine (BCAA)	**0.97 (d)**, 1.73 (m), 3.74 (m)	+0.43	0.10	-0.26	0.73	+0.44	**<0.001**
lysine	1.47 (m), 1.72 (m), **3.02 (m)**, 3.77 (t)	+0.06	0.06	-0.16	0.55	-0.80	**<0.001**
methionine	2.14 (s), 2.16 (m), **3.86 (dd)**	+0.58	0.11	+0.17	0.37	-	-
methylhistidine	3.24 (dd**), 3.70 (s)**, 7.10 (s), 7.90 (s)	+0.72	0.12	+0.32	0.22	-	-
NAD+	4.22, 4.39, 4.53, 6.02, 6.08, 8.16, 8.18, 8.41, **8.47 (s)**, 8.83 (d), 9.13, 9.32	+0.18	0.16	-0.45	**0.02**	-1.16	**<0.001**

[OFFICIAL]

Table 1. (continued)

Metabolites	δ ¹H ppm and multiplicity	Log_2FC (1 mM)	P-value (1 mM)	Log_2FC (10 mM)	P-value (10 mM)	Log_2FC (25 mM)	P-value (25 mM)
N, N-dimethylglycine	**2.92 (s)**, 3.72 (s)	+0.24	0.11	+0.61	0.10	-	-
N6-acetyllysine	1.41 (m), 1.90 (m), **3.74 (t)**	-0.05	0.78	+0.08	0.08	-	-
phenylalanine	3.13 (dd), 3.29 (dd), 3.97 (m), 7.33 (m), **7.38 (m)**, 7.43 (m)	+0.08	0.05	-0.27	**0.01**	-	-
phosphocholine	3.24 (s), **3.59 (m)**, 4.15 (m)	+0.65	0.09	+0.33	0.19	-0.86	**<0.001**
pyroglutamate	2.39 (m), **2.50 (m)**, 4.17 (dd)	+0.14	0.17	-0.32	0.10	-	-
pyruvate	2.36 (s)	+0.16	**0.06**	-0.53	**0.005**	-1.43	**<0.001**
succinate	**2.41 (s)**	+0.08	0.09	-0.62	0.058	+0.26	0.07
taurine	**3.26 (t)**, 3.42 (t)	+0.33	0.08	+0.56	0.19	+0.50	**0.023**
threonine	**1.32 (d)**, 3.58 (d), 4.24 (m)	-0.06	0.41	-0.87	**0.039**	-0.44	**0.021**
2-hydroxybutyrate	0.91 (t), **1.64 (m)**, 1.74 (m), 4.01 (m)	+0.48	0.74	+1.15	**0.04**	-	-
valine (BCAA)	**0.99 (d)**, 1.04 (d), 2.28 (m), 3.57 (d)	+0.56	0.07	-0.06	0.66	-0.12	0.83

[OFFICIAL]

Supplementary Table 2 – Quantitative comparison of the metabolites identified in HepG2 cell supernatants treated with 1 mM, 10 mM, and 25 mM (n = 5/group) H_2O_2 for 2 h. All $\log_2$ fold changes and *p*-values are compared to control samples. Resonances in bold were used to quantify metabolite concentrations. Statistical significance ($\log_2$ fold change > 1 or < - 1) is indicated in bold ($p < 0.05$).

Metabolites	δ ¹H ppm and multiplicity	Log_2FC (1 mM)	P-value (1 mM)	Log_2FC (10 mM)	P-value (10 mM)	Log_2FC (25 mM)	P-value (25 mM)
acetate	**1.93 (s)**	+1.03	**<0.001**	+1.78	**<0.001**	+2.00	**<0.001**
alanine	**1.48 (d)**, 3.78 (q)	+0.11	**0.03**	-0.34	**0.002**	-2.98	**<0.001**
arginine	1.91 (m), 1.64 (m), 3.24 (t), **3.78 (t)**	-0.03	0.80	+0.06	0.26	-0.29	0.67
ADP	**4.22 (m)**, 4.29 (m), 4.59 (t), 6.13 (d), 8.26 (s), 8.52 (s)	-0.004	0.46	+0.66	**0.01**	-	-
betaine	3.27 (s), **3.89 (s)**	-0.02	0.94	+0.08	0.10	+0.95	**<0.001**
creatine	**3.04 (s)**, 3.94 (s)	-0.03	0.77	-0.14	0.05	-	-
ethanol	**1.20 (t),** 3.65 (q)	+1.26	**<0.001**	+1.01	**<0.001**	-	-
formate	**8.46 (s)**	+0.16	0.36	+1.23	**<0.001**	**-1.88**	**<0.001**
glucose	3.23 (dd), 3.39 (m), 3.49 (t), 3.72 (dd), 3.81 (dd), 3.88 (dd), 4.63 (d), **5.24 (d)**	-0.04	0.94	+0.08	0.10	+2.17	**<0.001**
glutamate	2.11 (m), 2.33 (m), **3.75 (m)**	-0.04	0.98	+0.08	0.08	+0.72	**<0.001**
glutamine	2.13 (m), **2.44 (m)**, 3.78 (m)	-0.04	0.75	+0.03	0.60	+0.92	**<0.001**
glycine	**3.58 (s)**	+0.05	0.50	+0.08	0.29	-0.07	0.06
isoleucine (BCAA)	0.96 (t), **1.02 (d)**, 1.46 (m)	-0.03	0.85	+0.04	0.46	+0.58	**<0.001**
lactate	1.32 (d), **4.12 (q)**	-0.10	0.10	-1.39	**<0.001**	-4.70	**<0.001**
leucine (BCAA)	**0.97 (d)**, 1.73 (m), 3.74 (m)	-0.06	0.63	-0.002	0.90	+0.93	**<0.001**
lysine	1.47 (m), 1.72 (m), **3.02 (m)**, 3.77 (t)	-0.004	0.46	+0.05	0.29	+0.30	**<0.001**
methionine	2.14 (s), 2.16 (m), **3.86 (t)**	-0.07	0.73	-0.32	0.68	+0.78	**<0.001**
methylhistidine	3.24 (dd**), 3.70 (s)**, 7.10 (s), 7.90 (s)	-0.05	0.70	+0.09	0.08	+0.85	**<0.001**
N, N-dimethylglycine	**2.92 (s)**, 3.72 (s)	+0.19	0.34	+0.22	0.26	-1.06	**<0.001**
phenylalanine	3.13 (dd), 3.29 (dd), 3.97 (m), 7.33 (m), **7.38 (m)**, 7.43 (m)	-0.02	0.66	+0.14	**0.02**	+1.98	**<0.001**

[OFFICIAL]

Table 2. (continued)

**Metabolites**	**δ ^{1}H ppm and multiplicity**	**Log_2FC (1 mM)**	**P-value (1 mM)**	**Log_2FC (10 mM)**	**P-value (10 mM)**	**Log_2FC (25 mM)**	**P-value (25 mM)**
pyroglutamate	2.39 (m), **2.50 (m)**, 4.17 (dd)	-0.10	0.29	+0.02	0.12	+1.17	**<0.001**
pyruvate	2.36 (s)	-0.23	**0.02**	-1.90	**<0.001**	-7.80	**<0.001**
threonine	**1.32 (d)**, 3.58 (m), 4.24 (m)	-0.23	**0.02**	-1.90	**<0.001**	-4.43	**<0.001**
tyrosine	**3.06 (dd)**, 3.96 (m), 6.90 (d), 7.21 (m)	+0.03	0.33	+0.07	0.11	+1.36	**<0.001**
succinate	**2.41 (s)**	-0.05	0.51	0.07	0.19	+0.89	**<0.001**
valine (BCAA)	**0.99 (d)**, 1.04 (d), 2.28 (m), 3.57 (d)	-0.04	0.78	+0.01	0.97	-1.32	**<0.001**

Supplementary Table 3 – Quantitative comparison of metabolites found in rifampicin (+R) treated versus untreated (-R) HepG2 cell extracts and supernatants exposed to 25 mM H_2O_2 for 2 h (n = 5). All $\log_2$ fold changes and *p*-values in bold indicate statistical significance compared to controls ($\log_2$ fold change > 1 or < - 1 and $p < 0.05$).

Metabolites	Log_2FC Cell extracts (+R)	P-value Cell extracts (+R)	Log_2FC Cell extracts (-R)	P-value Cell extracts (-R)	Log_2FC Supernatants (+R)	P-value Supernatants (+R)	Log_2FC Supernatants (-R)	P-value Supernatants (-R)
acetate	**+1.29**	**0.05**	**-1.33**	**<0.001**	**+1.78**	**<0.001**	**-2.0**	**<0.001**
alanine	**-0.96**	**0.01**	**-2.97**	**<0.001**	-0.70	**<0.001**	**-2.98**	**<0.001**
ADP	-0.64	**0.03**	**-1.17**	**<0.001**	-	-	-	-
betaine	**+1.17**	0.08	+0.83	**<0.001**	-0.01	0.15	+0.95	**<0.001**
citrate	-0.01	0.63	**+1.12**	**<0.001**	-	-	-	-
creatine	+0.80	**0.01**	-0.90	**0.02**	-0.26	**0.02**	-0.39	**0.01**
creatine phosphate	**-2.06**	**<0.001**	**-1.91**	**<0.001**	-	-	-	-
formate	+0.15	0.63	**+1.15**	**<0.001**	**+1.61**	**<0.001**	**-1.88**	**<0.001**
fructose	+0.43	0.09	**+2.74**	**<0.001**	-0.003	0.21	**+2.2**	**<0.001**
glucose	**+1.33**	**0.08**	**+1.90**	**<0.001**	+0.01	0.10	**+2.17**	**<0.001**
glutamate	-0.28	0.85	**-1.23**	**<0.001**	+0.02	0.07	+0.72	**<0.001**
glutamine	**-1.15**	**0.01**	**-1.18**	**<0.001**	-0.06	0.24	**-0.67**	**<0.001**
glutathione	**+1.19**	**<0.001**	**+1.05**	**0.01**	-	-	-	-
glycine	-0.51	0.11	**-1.18**	**<0.001**	+0.04	0.16	-0.07	0.06
isoleucine (BCAA)	-0.08	0.36	-0.13	0.06	-0.04	0.59	+0.58	**<0.001**
lactate	**-1.13**	**0.02**	**-1.97**	**<0.001**	**-1.87**	**<0.001**	**-4.70**	**<0.001**
leucine (BCAA)	-0.33	0.74	+0.44	**<0.001**	-0.08	0.15	+0.93	**<0.001**
lysine	-0.07	0.97	-0.80	**<0.001**	-0.05	0.88	+0.30	**<0.001**
methionine	+0.23	0.13	-	-	-0.06	**<0.001**	+0.78	**<0.001**
methylhistidine	+0.63	0.09	-	-	+0.02	0.06	+0.85	**<0.001**
NAD+	**-1.24**	**<0.001**	**-1.16**	**<0.001**	-	-	-	-
phenylalanine	-0.37	**<0.001**	-	-	+0.04	0.05	**+1.98**	**<0.001**

[OFFICIAL]

Table 3. (continued)

Metabolites	Log_2FC Cell extracts (+R)	P-value Cell extracts (+R)	Log_2FC Cell extracts (-R)	P-value Cell extracts (-R)	Log_2FC Supernatants (+R)	P-value Supernatants (+R)	Log_2FC Supernatants (-R)	P-value Supernatants (-R)
phosphocholine	+0.53	0.07	-0.86	**<0.001**	-	-	-	-
pyroglutamate	-0.42	0.09	+0.40	0.10	+0.06	**0.01**	**+1.17**	**<0.001**
pyruvate	**-1.03**	**0.004**	**-1.43**	**<0.001**	**-3.08**	**<0.001**	-7.80	**<0.001**
succinate	-0.87	**0.003**	+0.26	**0.02**	+0.02	0.19	+0.89	**<0.001**
taurine	**+1.33**	**0.02**	+0.50	**<0.001**	-	-	-	-
threonine	**-1.27**	**0.03**	-0.44	**0.02**	**-3.08**	**<0.001**	**-4.43**	**<0.001**
2-hydroxybutyrate	**+1.33**	**<0.001**	+0.44	**0.01**	-	-	-	-
valine (BCAA)	-0.05	0.33	+0.12	0.83	-0.07	0.17	**-1.32**	**<0.001**

[OFFICIAL]
